# Crystal structure of β-d,l-psicose

**DOI:** 10.1107/S2056989015006623

**Published:** 2015-04-09

**Authors:** Tomohiko Ishii, Genta Sakane, Akihide Yoshihara, Kazuhiro Fukada, Tatsuya Senoo

**Affiliations:** a Department of Advanced Materials Science, Faculty of Engineering, Kagawa University, 2217-20 Hayashi-cho, Takamatsu, Kagawa 761-0396, Japan; b Department of Chemistry, Faculty of Science, Okayama University of Science, 1-1 Ridaicho, Kita-ku, Okayama 700-0005, Japan; c Rare Sugar Research Center, Faculty of Agriculture, Kagawa University, 2393 Ikenobe, Kagawa 761-0795, Japan; d Department of Applied Biological Science, Faculty of Agriculture, Kagawa University, 2393 Ikenobe, Kagawa 761-0795, Japan

**Keywords:** crystal structure, hydrogen bonding, racemic compound, rare sugar

## Abstract

The title compound, C_6_H_12_O_6_, a C-3 position epimer of fructose, was crystallized from an aqueous solution of equimolar mixture of d- and l-psicose (1,3,4,5,6-penta­hydroxy­hexan-2-one, *ribo*-2-hexulose, allulose), and it was confirmed that d-psicose (or l-psicose) formed β-pyran­ose with a ^2^ *C*_5_ (or ^5^ *C*_2_) conformation. In the crystal, an O—H⋯O hydrogen bond between the hy­droxy groups at the C-3 and C-2 positions connects homochiral mol­ecules into a column along the *b* axis. The columns are linked by other O—H⋯O hydrogen bonds between d- and l-psicose mol­ecules, forming a three-dimensional network. An intra­molecular O—H⋯O hydrogen bond is also observed. The cell volume of racemic β-d,l-psicose [763.21 (6) Å^3^] is almost the same as that of chiral β-d-psicose [753.06 Å^3^].

## Related literature   

For the crystal structure of the chiral β-d-psicose, see: Kwiecien *et al.* (2008 ▸); Fukada *et al.* (2010 ▸). For the synthesis of the chiral d-psicose, see: Itoh *et al.* (1995 ▸); Takeshita *et al.* (2000 ▸). For the synthesis of the chiral l-psicose, see: Takeshita *et al.* (1996 ▸).
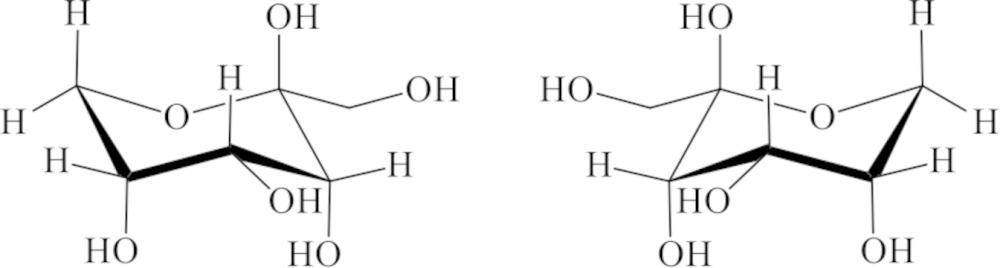


## Experimental   

### Crystal data  

C_6_H_12_O_6_
*M* *_r_* = 180.16
Orthorhombic, 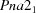

*a* = 11.2629 (5) Å
*b* = 5.3552 (3) Å
*c* = 12.6538 (6) Å
*V* = 763.21 (6) Å^3^
*Z* = 4
Cu *K*α radiation
μ = 1.25 mm^−1^
*T* = 296 K
0.10 × 0.10 × 0.10 mm

### Data collection  

Rigaku R-AXIS RAPID diffractometer
Absorption correction: multi-scan (*ABSCOR*; Higashi, 1995 ▸) *T*_min_ = 0.442, *T*_max_ = 0.883
12119 measured reflections
1400 independent reflections
1295 reflections with *F* ^2^ > 2σ(*F* ^2^)
*R*_int_ = 0.139

### Refinement  

*R*[*F* ^2^ > 2σ(*F* ^2^)] = 0.048
*wR*(*F* ^2^) = 0.102
*S* = 1.04
1400 reflections
116 parameters
1 restraint
H-atom parameters constrained
Δρ_max_ = 0.25 e Å^−3^
Δρ_min_ = −0.23 e Å^−3^
Absolute structure: Flack (1983 ▸), 666 Friedel pairs
Absolute structure parameter: 0.1 (4)

### 

Data collection: *RAPID-AUTO* (Rigaku, 2009 ▸); cell refinement: *RAPID-AUTO*; data reduction: *RAPID-AUTO*; program(s) used to solve structure: *SIR2011* (Burla *et al.*, 2012 ▸); program(s) used to refine structure: *SHELXL2013* (Sheldrick, 2015 ▸); molecular graphics: *CrystalStructure* (Rigaku, 2014 ▸); software used to prepare material for publication: *CrystalStructure*.

## Supplementary Material

Crystal structure: contains datablock(s) global, I. DOI: 10.1107/S2056989015006623/is5394sup1.cif

Structure factors: contains datablock(s) I. DOI: 10.1107/S2056989015006623/is5394Isup2.hkl

**Fig. 1.**
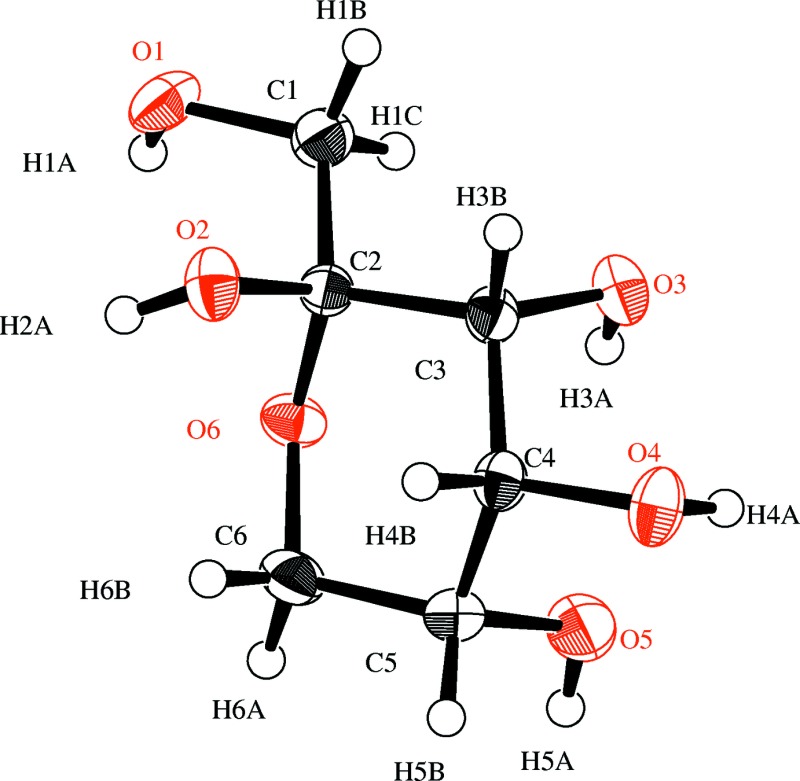
ORTEP view of the title compound with the atom-labeling scheme. The thermal ellipsoids of all non-hydrogen atoms are drawn at the 50% probability level. H atoms are shown as small spheres of arbitrary radius.

**Fig. 2.**
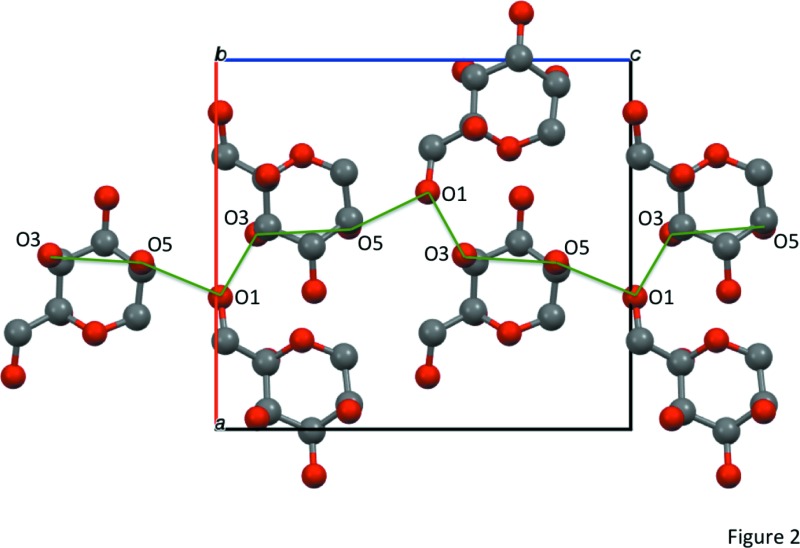
Part of the crystal structure of the title compound with hydrogen-bonding network represented as green solid lines, viewed down the b-axis. The hydrogen atoms are omitted for clarity.

**Fig. 3.**
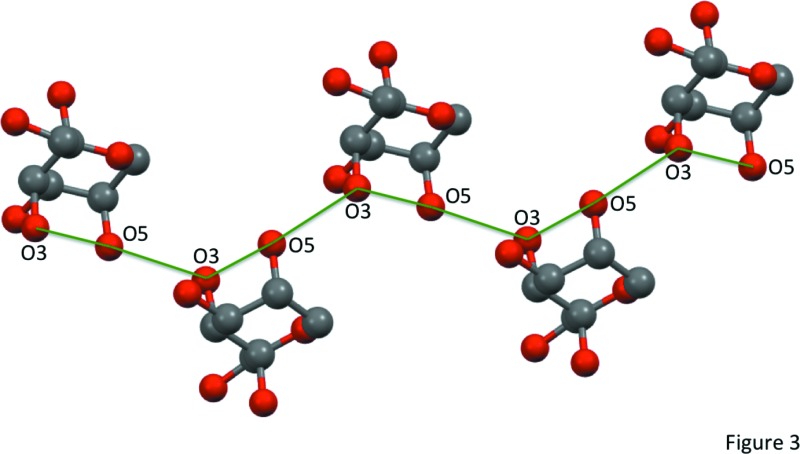
Part of the crystal structure of the chiral β-D-psicose (Fukada et al., 2010) with hydrogen-bonding network represented as green solid lines. The hydrogen atoms are omitted for clarity.

ORTEP . DOI: 10.1107/S2056989015006623/is5394fig1.tif
*ORTEP* view of the title compound with the atom-labeling scheme. The thermal ellipsoids of all non-hydrogen atoms are drawn at the 50% probability level. H atoms are shown as small spheres of arbitrary radius.

b . DOI: 10.1107/S2056989015006623/is5394fig2.tif
Part of the crystal structure of the title compound with hydrogen-bonding network represented as green solid lines, viewed down the *b*-axis. The hydrogen atoms are omitted for clarity.

d et al. . DOI: 10.1107/S2056989015006623/is5394fig3.tif
Part of the crystal structure of the chiral β-d-psicose (Fukada *et al.*, 2010) with hydrogen-bonding network represented as green solid lines. The hydrogen atoms are omitted for clarity.

CCDC reference: 1057484

Additional supporting information:  crystallographic information; 3D view; checkCIF report

## Figures and Tables

**Table 1:** Hydrogen-bond geometry (, )

*D*H*A*	*D*H	H*A*	*D* *A*	*D*H*A*
O1H1*A*O3^i^	0.82	1.91	2.715(3)	168
O2H2*A*O4^ii^	0.82	1.92	2.724(3)	166
O3H3*A*O2^iii^	0.82	2.20	2.874(3)	140
O3H3*A*O5	0.82	2.36	2.822(4)	117
O4H4*A*O6^iv^	0.82	2.14	2.829(3)	141
O5H5*A*O1^v^	0.82	1.94	2.746(4)	169

Symmetry codes: (i) 
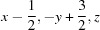
; (ii) 
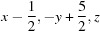
; (iii) 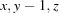; (iv) 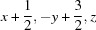; (v) 
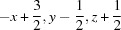
.

## supplementary crystallographic information

### S1. Comment

In the crystal of the title compound, the *D*- and *L*-molecules are located alternatively in *a-c* plane, so that the main hydrogen bonding networks can be created between *D*- and *L*-molecules. An additional hydrogen bonding between two *D*-molecules (and two *L*-molecules) are observed along to the *b*-axis (O3—H3A···O2^iii^; Table 1). The molecular structure of *D*-psicose (or *L*-psicose) is β-pyranose form with a ^2^C_5_ (or ^5^C_2_) conformation. Orientations of two OH groups at C-3 and C-5 positions are axial, therefore an intramolecular hydrogen bonding can be observed (O3—H3A···O5; 2.36 Å) [hereafter, (O3···O5)]. The intramolecular hydrogen bonding unit (O3···O5) shown in the racemic *D,L*-crystal has also observed in a chiral *D*-crystal (Fukada *et al.*, 2010). In the chiral one, one-dimensional hydrogen bonding chain, that is (O3···O5) -> (O3···O5) -> (O3···O5) ->···, can be observed by connecting through an another hydrogen bonding between two *D*-molecule units (O5—H5···O3). On the other hand in the case of the racemic one, the *L*-molecule (or *D*-molecule) plays as a role of a bridging between two adjacent intramolecular hydrogen bonding in *D*-molecule (or *L*-molecule) (O3···O5) units, that is (*D* O3···O5) -> (*L* O1) -> (*D* O3···O5) -> (*L* O1) -> ··· (or, (*L* O3···O5) -> (*D* O1) -> (*L* O3···O5) -> (*D* O1) -> ···). Concerning the intermolecular hydrogen bonding, there are four kinds of bondings are also observed between *D*- and *L*- psicose molecules (O1—H1A···O3 (*a*-axis), O2—H2A···O4, O4—H4A···O6 (*a*-axis), and O5—H5A···O1 (*c*-axis),). The cell volume of racemic β-*D,L*-psicose [763.21 (6) Å^3^ at r.t.] is almost the same as that of chiral β-*D*-psicose [753.06 Å^3^ at r.t.].

### S2. Experimental

*D*-Psicose was prepared from *D*-fructose by enzymatic epimerization using *D*-tagatose 3-epimerase (Itoh *et al.*, 1995; Takeshita *et al.*, 2000). *L*-Psicose was prepared from allitol by microbial oxidation using Gluconobacter frateurii IFO 3254 (Takeshita *et al.*, 1996). *D*-Psicose and *L*-psicose were mixed in equal amount and dissolved in hot water to give 60, 65, 70, 75, and 80 wt% solution. And these samples were kept at 10, 20, and 30 °C. After one day, small crystals were obtained in 65, 70, 75, and 80 wt% solution at 10, 20, and 30 °C.

### S3. Refinement

H atoms bounded to methine-type C (H3B, H4B, H5B) were positioned geometrically and refined using a riding model with C—H = 0.98 Å and *U*_iso_(H) = 1.2*U*_eq_(C). H atoms bounded to methylene-type C (H1B, H1C, H6A, H6B) were positioned geometrically and refined using a riding model with C—H = 0.97 Å and *U*_iso_(H) = 1.2*U*_eq_(C). H atoms bounded to O (H1A, H2A, H3A, H4A, H5A) were positioned geometrically and refined using a riding model with O—H = 0.82 Å and *U*_iso_(H) = 1.2*U*_eq_(O), allowing for free rotation of the OH groups.

### Crystal data

**Table:**

C_6_H_12_O_6_	*D*_x_ = 1.568 Mg m^−^^3^
*M**_r_* = 180.16	Cu *K*α radiation, λ = 1.54187 Å
Orthorhombic, *P**n**a*2_1_	Cell parameters from 5584 reflections
*a* = 11.2629 (5) Å	θ = 3.5–68.5°
*b* = 5.3552 (3) Å	µ = 1.25 mm^−^^1^
*c* = 12.6538 (6) Å	*T* = 296 K
*V* = 763.21 (6) Å^3^	Block, colorless
*Z* = 4	0.10 × 0.10 × 0.10 mm
*F*(000) = 384.00	

### Data collection

**Table:**

Rigaku R-AXIS RAPID diffractometer	1295 reflections with *F*^2^ > 2σ(*F*^2^)
Detector resolution: 10.000 pixels mm^-1^	*R*_int_ = 0.139
ω scans	θ_max_ = 68.2°, θ_min_ = 7.0°
Absorption correction: multi-scan (*ABSCOR*; Higashi, 1995)	*h* = −13→13
*T*_min_ = 0.442, *T*_max_ = 0.883	*k* = −6→6
12119 measured reflections	*l* = −15→15
1400 independent reflections	

### Refinement

**Table:**

Refinement on *F*^2^	H-atom parameters constrained
*R*[*F*^2^ > 2σ(*F*^2^)] = 0.048	*w* = 1/[σ^2^(*F*_o_^2^) + (0.0359*P*)^2^] where *P* = (*F*_o_^2^ + 2*F*_c_^2^)/3
*wR*(*F*^2^) = 0.102	(Δ/σ)_max_ < 0.001
*S* = 1.04	Δρ_max_ = 0.25 e Å^−^^3^
1400 reflections	Δρ_min_ = −0.23 e Å^−^^3^
116 parameters	Extinction correction: *SHELXL*
1 restraint	Extinction coefficient: 0.039 (3)
Primary atom site location: structure-invariant direct methods	Absolute structure: Flack (1983), 666 Friedel pairs
Secondary atom site location: difference Fourier map	Absolute structure parameter: 0.1 (4)
Hydrogen site location: inferred from neighbouring sites	

### Special details

**Table:**

Geometry. ENTER SPECIAL DETAILS OF THE MOLECULAR GEOMETRY
Refinement. Refinement was performed using all reflections. The weighted *R*-factor (*wR*) and goodness of fit (*S*) are based on *F*^2^. *R*-factor (gt) are based on *F*. The threshold expression of *F*^2^ > 2.0 σ(*F*^2^) is used only for calculating *R*-factor (gt).

### Fractional atomic coordinates and isotropic or equivalent isotropic displacement parameters (Å^2^)

**Table:**

	*x*	*y*	*z*	*U*_iso_*/*U*_eq_	
O1	0.6428 (2)	1.0475 (5)	0.0101 (2)	0.0321 (7)	
O2	0.8138 (2)	1.3225 (4)	0.1244 (2)	0.0283 (6)	
O3	0.9712 (2)	0.7407 (4)	0.0986 (2)	0.0278 (6)	
O4	1.12635 (19)	0.9941 (5)	0.2360 (2)	0.0306 (7)	
O5	0.9520 (3)	0.6941 (5)	0.3201 (2)	0.0368 (7)	
O6	0.75926 (19)	0.9487 (5)	0.2068 (2)	0.0243 (6)	
C1	0.7610 (3)	0.9600 (8)	0.0218 (3)	0.0275 (8)	
C2	0.8199 (3)	1.0614 (6)	0.1199 (3)	0.0211 (7)	
C3	0.9525 (3)	0.9956 (6)	0.1238 (3)	0.0223 (7)	
C4	1.0049 (3)	1.0665 (7)	0.2306 (2)	0.0233 (8)	
C5	0.9337 (3)	0.9564 (7)	0.3210 (3)	0.0266 (8)	
C6	0.8044 (3)	1.0258 (7)	0.3083 (3)	0.0289 (8)	
H1B	0.80695	1.00892	−0.03968	0.0330*	
H1C	0.76042	0.77904	0.02497	0.0330*	
H1A	0.59772	0.95756	0.04384	0.0386*	
H2A	0.75043	1.36467	0.15074	0.0340*	
H3A	0.92832	0.65324	0.13561	0.0333*	
H3B	0.99273	1.0959	0.06985	0.0268*	
H4B	1.00147	1.24872	0.23691	0.0280*	
H4A	1.1313	0.84167	0.23198	0.0367*	
H5A	0.92192	0.63218	0.37294	0.0442*	
H5B	0.96322	1.0246	0.38792	0.0319*	
H6A	0.75828	0.94707	0.36378	0.0347*	
H6B	0.79566	1.20525	0.31544	0.0347*	

### Atomic displacement parameters (Å^2^)

**Table:**

	*U*^11^	*U*^22^	*U*^33^	*U*^12^	*U*^13^	*U*^23^
O1	0.0232 (14)	0.0431 (18)	0.0301 (13)	−0.0019 (11)	−0.0058 (11)	0.0126 (12)
O2	0.0238 (12)	0.0228 (13)	0.0384 (14)	0.0010 (9)	0.0057 (11)	0.0025 (13)
O3	0.0252 (12)	0.0240 (13)	0.0340 (14)	0.0013 (10)	0.0068 (10)	−0.0020 (11)
O4	0.0193 (12)	0.0265 (14)	0.0459 (17)	0.0004 (10)	−0.0022 (10)	0.0013 (12)
O5	0.0475 (17)	0.0309 (15)	0.0322 (15)	0.0064 (12)	0.0101 (11)	0.0101 (12)
O6	0.0230 (12)	0.0297 (14)	0.0203 (11)	−0.0057 (10)	0.0027 (10)	0.0002 (12)
C1	0.025 (2)	0.032 (2)	0.0254 (19)	−0.0001 (15)	−0.0001 (14)	0.0024 (18)
C2	0.0211 (17)	0.0228 (17)	0.0195 (16)	0.0012 (12)	0.0034 (14)	0.0043 (16)
C3	0.0197 (19)	0.0233 (17)	0.0239 (17)	0.0009 (12)	0.0038 (14)	0.0042 (14)
C4	0.0189 (17)	0.025 (2)	0.0265 (19)	0.0009 (13)	0.0000 (13)	0.0006 (15)
C5	0.030 (2)	0.030 (2)	0.0201 (17)	0.0027 (14)	−0.0011 (15)	−0.0001 (15)
C6	0.0272 (18)	0.039 (2)	0.0209 (17)	0.0006 (15)	0.0055 (15)	−0.0002 (15)

### Geometric parameters (Å, º)

**Table:**

O1—C1	1.419 (4)	O1—H1A	0.820
O2—C2	1.401 (4)	O2—H2A	0.820
O3—C3	1.418 (4)	O3—H3A	0.820
O4—C4	1.423 (4)	O4—H4A	0.820
O5—C5	1.419 (4)	O5—H5A	0.820
O6—C2	1.428 (4)	C1—H1B	0.970
O6—C6	1.442 (4)	C1—H1C	0.970
C1—C2	1.509 (5)	C3—H3B	0.980
C2—C3	1.535 (5)	C4—H4B	0.980
C3—C4	1.523 (5)	C5—H5B	0.980
C4—C5	1.516 (5)	C6—H6A	0.970
C5—C6	1.512 (5)	C6—H6B	0.970
			
C2—O6—C6	113.3 (2)	C4—O4—H4A	109.469
O1—C1—C2	112.3 (3)	C5—O5—H5A	109.469
O2—C2—O6	111.6 (3)	O1—C1—H1B	109.152
O2—C2—C1	111.8 (3)	O1—C1—H1C	109.152
O2—C2—C3	106.0 (3)	C2—C1—H1B	109.145
O6—C2—C1	105.7 (3)	C2—C1—H1C	109.145
O6—C2—C3	110.1 (3)	H1B—C1—H1C	107.867
C1—C2—C3	111.8 (3)	O3—C3—H3B	107.581
O3—C3—C2	111.0 (3)	C2—C3—H3B	107.578
O3—C3—C4	112.5 (3)	C4—C3—H3B	107.580
C2—C3—C4	110.4 (3)	O4—C4—H4B	107.757
O4—C4—C3	110.3 (3)	C3—C4—H4B	107.761
O4—C4—C5	111.5 (3)	C5—C4—H4B	107.767
C3—C4—C5	111.6 (3)	O5—C5—H5B	109.099
O5—C5—C4	107.5 (3)	C4—C5—H5B	109.103
O5—C5—C6	112.5 (3)	C6—C5—H5B	109.093
C4—C5—C6	109.5 (3)	O6—C6—H6A	109.357
O6—C6—C5	111.4 (3)	O6—C6—H6B	109.357
C1—O1—H1A	109.471	C5—C6—H6A	109.358
C2—O2—H2A	109.471	C5—C6—H6B	109.358
C3—O3—H3A	109.470	H6A—C6—H6B	107.992
			
C2—O6—C6—C5	−60.7 (3)	C1—C2—C3—C4	−171.7 (2)
C6—O6—C2—O2	−58.2 (3)	O3—C3—C4—O4	52.5 (3)
C6—O6—C2—C1	−179.9 (2)	O3—C3—C4—C5	−72.0 (3)
C6—O6—C2—C3	59.2 (3)	C2—C3—C4—O4	177.1 (2)
O1—C1—C2—O2	−53.5 (4)	C2—C3—C4—C5	52.6 (3)
O1—C1—C2—O6	68.1 (3)	O4—C4—C5—O5	−54.2 (3)
O1—C1—C2—C3	−172.1 (2)	O4—C4—C5—C6	−176.7 (2)
O2—C2—C3—O3	−168.2 (2)	C3—C4—C5—O5	69.6 (3)
O2—C2—C3—C4	66.4 (3)	C3—C4—C5—C6	−52.9 (3)
O6—C2—C3—O3	71.0 (3)	O5—C5—C6—O6	−63.8 (4)
O6—C2—C3—C4	−54.4 (3)	C4—C5—C6—O6	55.7 (4)
C1—C2—C3—O3	−46.2 (4)		

### Hydrogen-bond geometry (Å, º)

**Table:**

*D*—H···*A*	*D*—H	H···*A*	*D*···*A*	*D*—H···*A*
O1—H1*A*···O3^i^	0.82	1.91	2.715 (3)	168
O2—H2*A*···O4^ii^	0.82	1.92	2.724 (3)	166
O3—H3*A*···O2^iii^	0.82	2.20	2.874 (3)	140
O3—H3*A*···O5	0.82	2.36	2.822 (4)	117
O4—H4*A*···O6^iv^	0.82	2.14	2.829 (3)	141
O5—H5*A*···O1^v^	0.82	1.94	2.746 (4)	169

Symmetry codes: (i) *x*−1/2, −*y*+3/2, *z*; (ii) *x*−1/2, −*y*+5/2, *z*; (iii) *x*, *y*−1, *z*; (iv) *x*+1/2, −*y*+3/2, *z*; (v) −*x*+3/2, *y*−1/2, *z*+1/2.

## References

[bb1] Burla, M. C., Caliandro, R., Camalli, M., Carrozzini, B., Cascarano, G. L., Giacovazzo, C., Mallamo, M., Mazzone, A., Polidori, G. & Spagna, R. (2012). *J. Appl. Cryst.* **45**, 357–361.

[bb2] Flack, H. D. (1983). *Acta Cryst.* A**39**, 876–881.

[bb3] Fukada, K., Ishii, T., Tanaka, K., Yamaji, M., Yamaoka, Y., Kobashi, K. & Izumori, K. (2010). *Bull. Chem. Soc. Jpn*, **83**, 1193–1197.

[bb4] Higashi, T. (1995). *ABSCOR*. Rigaku Corporation, Tokyo, Japan.

[bb5] Itoh, H., Sato, T. & Izumori, K. (1995). *J. Ferment. Bioeng.* **80**, 101–103.

[bb6] Kwiecien, A., Slepokura, K. & Lis, T. (2008). *Carbohydr. Res.* **343**, 2336–2339. 10.1016/j.carres.2008.05.012 18547550

[bb7] Rigaku (2009). *RAPID-AUTO*. Rigaku Corporation, Tokyo, Japan.

[bb8] Rigaku (2014). *CrystalStructure*. Rigaku Corporation, Tokyo, Japan.

[bb9] Sheldrick, G. M. (2015). *Acta Cryst.* C**71**, 3–8.

[bb10] Takeshita, K., Shimonishi, T. & Izumori, K. (1996). *J. Ferment. Bioeng.* **81**, 212–215.

[bb11] Takeshita, K., Suga, A., Takada, G. & Izumori, K. (2000). *J. Biosci. Bioeng.* **90**, 453–455. 10.1016/s1389-1723(01)80018-9 16232889

