# Supplementary figures and images for: Crystal structure of β-d,l-psicose
Source: Acta Crystallogr E Crystallogr Commun. 2015 Apr 9;71(Pt 5):o289–90. doi: 10.1107/S2056989015006623 (PMC4420085; doi:10.1107/S2056989015006623)

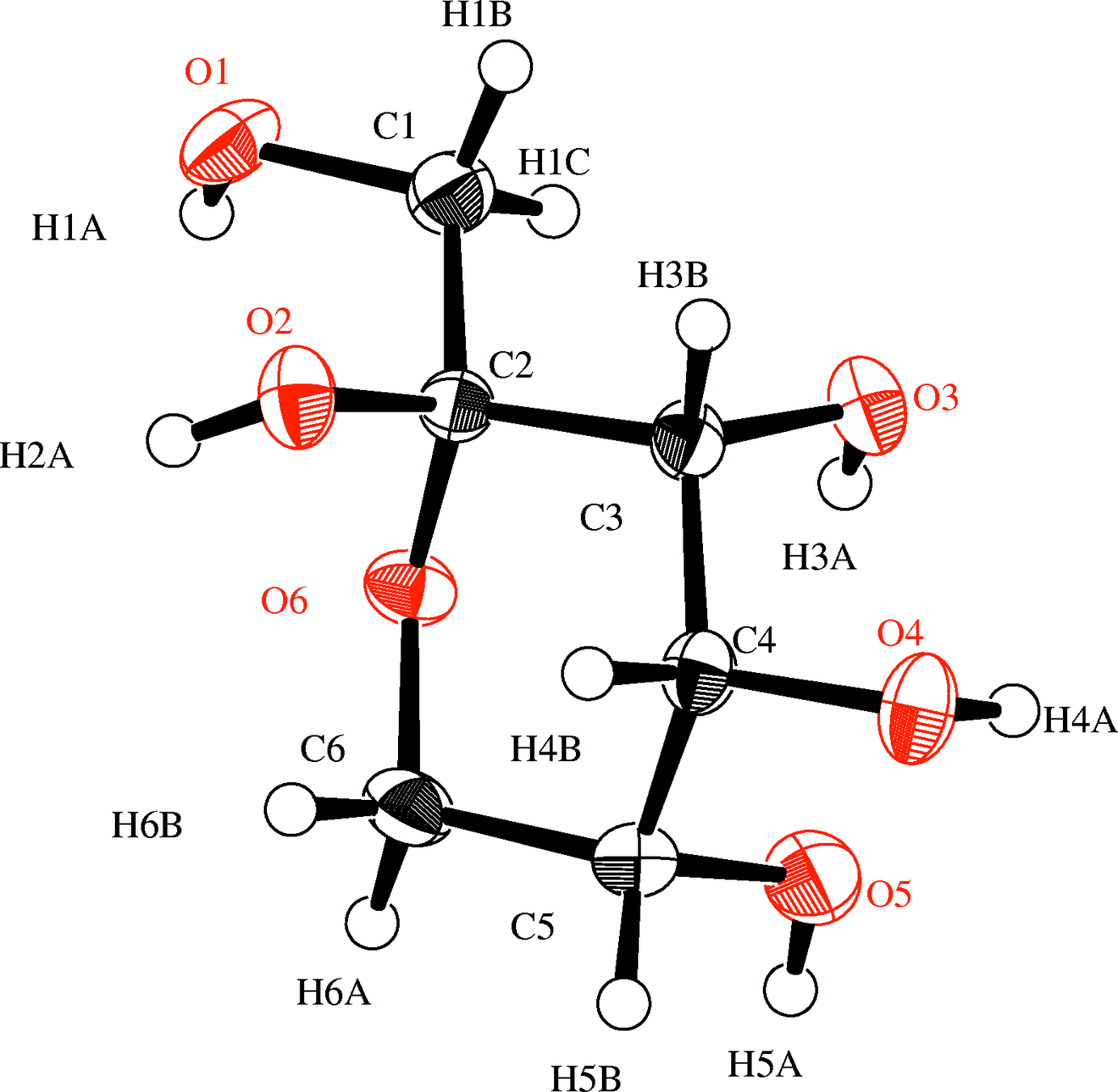

Supplement: Supplementary file 3 [file e-71-0o289-fig1.tif]

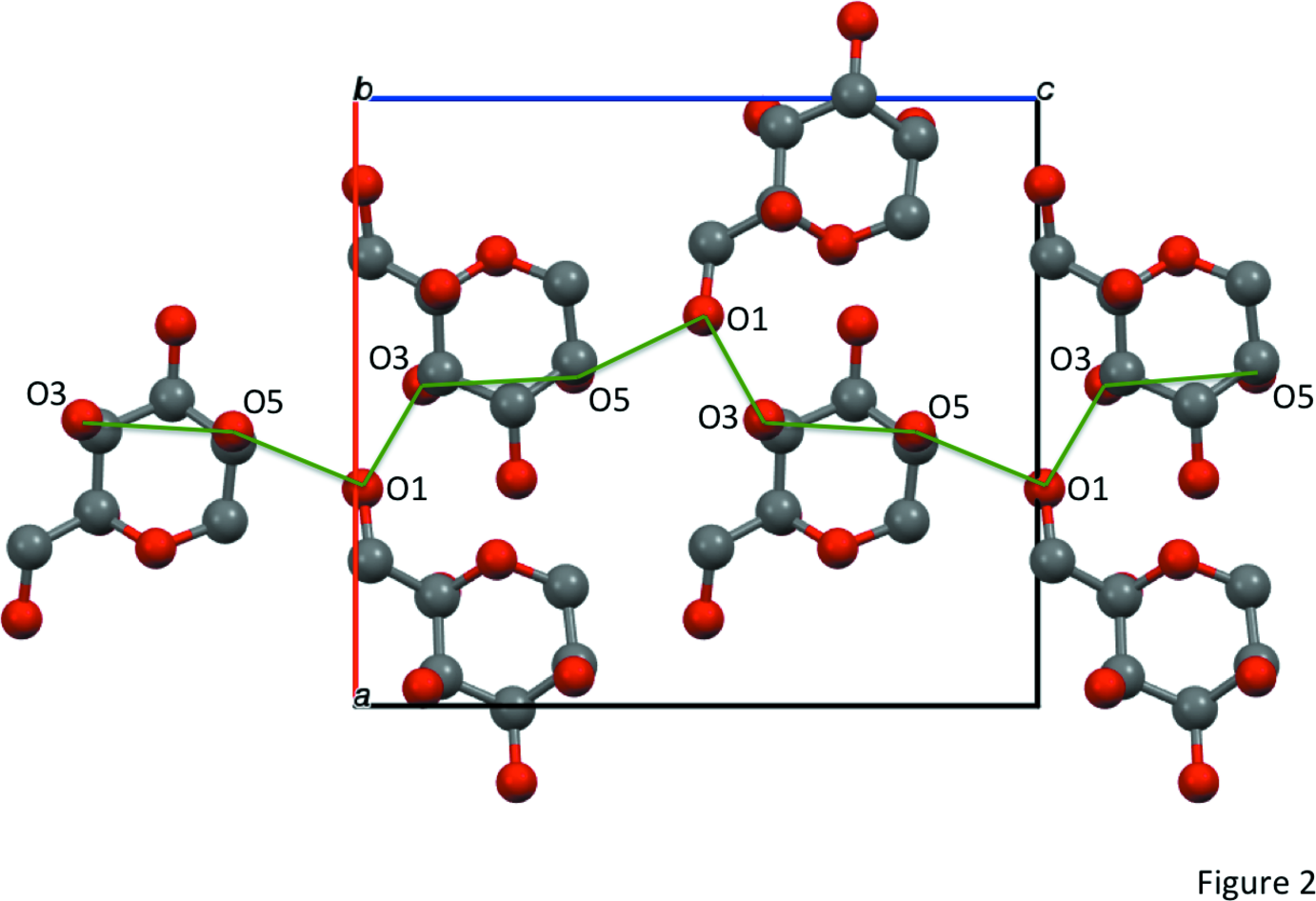

Supplement: Supplementary file 4 [file e-71-0o289-fig2.tif]

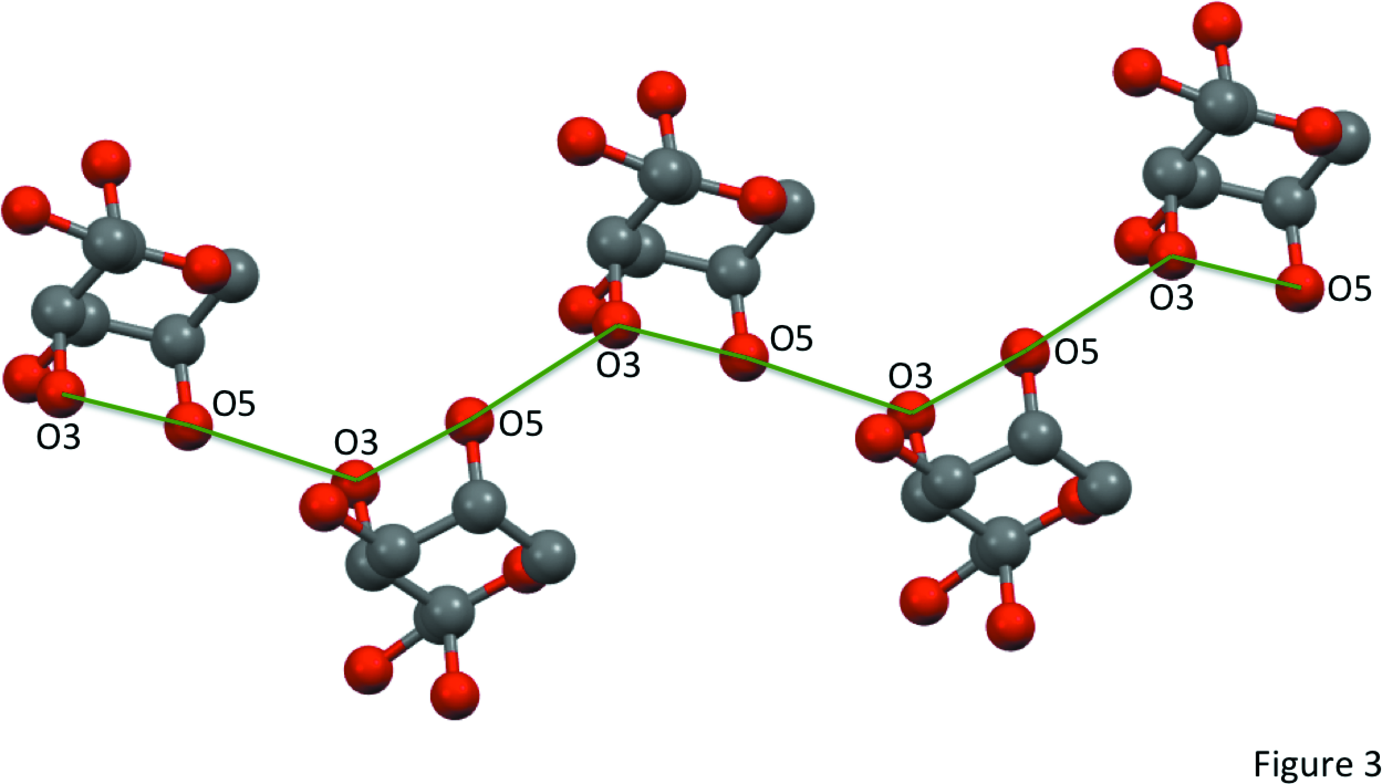

Supplement: Supplementary file 5 [file e-71-0o289-fig3.tif]
